# Bridging Ecological Rationality, Embodied Emotion, and Neuroeconomics: Insights From the Somatic Marker Hypothesis

**DOI:** 10.3389/fpsyg.2020.01028

**Published:** 2020-06-03

**Authors:** Fuming Xu, Peng Xiang, Long Huang

**Affiliations:** ^1^School of Education Science, Nanning Normal University, Nanning, China; ^2^School of Law, Nanjing University of Finance and Economics, Nanjing, China; ^3^School of Humanities and Management, Wannan Medical College, Wuhu, China; ^4^School of Psychology, Jiangxi Normal University, Nanchang, China

**Keywords:** somatic marker hypothesis, ecological rationality, embodied emotion, neuroeconomics, decision-making

## Abstract

The somatic marker hypothesis (SMH) has been utilized to demonstrate the role of emotion and somatic state in decision-making under uncertainty over the past two decades. Despite some debate, the SMH has provided not only a neurobiological framework for understanding emotion and decision-making but also a good empirical support for ecological rationality and embodied emotion. Unlike the traditional maximizing rationality and bounded satisficing rationality, the ecological rationality stresses that emotions should be brought to the decision-making process. The embodied emotion furthermore emphasizes that emotions are embodied in the body and the brain. On the other hand, behavioral decision-making has spawned many new interdisciplines, including neuroeconomics. In this case, the SMH could act as a bridge to translate the ecological rationality and the embodied emotion into emerging neuroeconomics. Thus, this mini-review article aims to propose an integrated framework for introducing ecological rationality and embodied emotion into the field of neuroeconomics by virtue of insights from the SMH.

## Introduction

Traditionally, researchers in the social science field, especially economists, have held the view that individual decision-making is the pursuit of utility maximization and regarded the principles of logic, probability theory, and game theory as axioms of judgment and decision-making. According to these researchers, decision-makers are assumed to be completely rational. However, it has been well documented that the empirical findings based on these assumptions could not provide firmly grounded inferences about the decision behavior in the real world. Although the normative model of rational decision contributed to the birth of behavioral decision-making, its central hypothesis, namely, utility maximization of the rational economic man, is derived from the synchronous axiomatization of expected utility and subjective probability rather than empirical evidence (Gigerenzer and Selten, 2002). With the advent of behavioral decision-making, the absoluteness and the predictability of decision-utility maximization have been challenged by increasing research. The initial challenges come from the “Allais Paradox,” proposed in 1953, and the “Ellsberg Paradox,” proposed in 1961. These two paradoxes refuted the cancelation rule of rational decision. What is more, Amos Tversky and Daniel Kahneman identified a series of heuristics and biases which could systematically violate the underlying normative principles of rational decision (Tversky and Kahneman, 1974; Kahneman, 2011). The framing effect, for instance, proposed by Tversky and Kahneman (1981), has directly demolished the invariability rule of rational decision. In addition to the above-mentioned empirical challenges, the validity of decision-utility maximization was also questioned by the theoretical progresses, especially Herbert Simon’s notions of bounded rationality and satisficing rule (Simon, 1997).

Research in the field of decision-making was once dominated by the hypothesis of the rational economic man. Under this hypothesis, decision-makers are described as perfectly rational calculators who aim at utility maximization. This assumption is highly dubious, however. According to Simon (1955, 1956), it is impossible for decision-makers to arrive at utility-maximizing decisions in the real world since they have no sufficient resources (e.g., time and knowledge) and cognitive capability required for rational decision-making. Therefore, it is very common that individual decision-making diverges from the predictions based on the model of utility-maximizing decision. Given that, Simon introduced the concept of bounded rationality. According to Simon, human rationality is bounded by both internal (mental) and external (environmental) constraints, and hence the idealized conditions of perfect rationality assumed by models of the economic man are unreachable. Simon further stated that human decision behavior “is shaped by a scissors whose two blades are the structure of task environments and the computational capabilities of the actor” (Simon, 1956, 1997). These two “blades” imply that the actor with limited computational capabilities could utilize the structure characteristics of task environments to achieve a “satisficing” choice. Clearly, Simon’s scissors metaphor emphasizes the importance of the match between the limited computational capabilities and the structures of the environment. By contrast, the idea of absolute rationality not only overestimates the computational capabilities of the decision-maker but also disregards the environment characteristics that the decision-maker is facing. However, what Simon in effect argued is that the human decision process can only be understood in terms of two scissor blades: mind and environment (Simon, 1997).

In the following section, we first summarize the basic ideas of ecological rationality, the somatic marker hypothesis (SMH), and embodied emotion. Second, similar notions among them are briefly discussed. Third, we argue that the SMH can serve as a bridge for introducing key concepts of ecological rationality and embodied emotion into the burgeoning neuroeconomic research. For this propose, an integrative framework is finally presented. All in all, the purpose of this review article is to demonstrate that the SMH can bridge ecological rationality, embodied emotion, and neuroeconomics and accordingly to construct an integrated framework for applying ecological rationality and embodied emotion to neuroeconomics by virtue of core insights from the SMH.

## Ecological Rationality and Somatic Marker Hypothesis

Following the above-mentioned pioneering work of Simon, Gerd Gigerenzer (2000) further claimed that sound reasoning could be achieved *via* simple heuristics that do not observe the rules of logic and probability (Gigerenzer and Todd, 1999). Based on Simon’s notion of bounded rationality, Gigerenzer (2000) proposed the concepts of ecological rationality and social rationality. The ecological rationality view emphasizes that cognitive ability and environmental structure are equally important and indispensable in the process of judgment and decision-making. According to the view of ecological rationality, decision-making is just a progress wherein the decision-maker makes full use of environmental information to reach well adaptability (Gigerenzer and Todd, 1999; Gigerenzer, 2000). In line with this view, research about ecological rationality has related the brain of the decision-maker with the past and the present environments and explored the information structure of the environment, the structure of heuristics, as well as the match of both (Gigerenzer and Selten, 2002; Gigerenzer and Gaissmaier, 2011).

In addition to the heuristic cognitive strategies, ecological rationality also acknowledges the significance of various non-cognitive heuristic strategies, e.g., emotions and social norms. Particularly, ecological rationality puts a great emphasis on the functions that humans gain from the process of adapting to their surroundings (Gigerenzer, 2000). Emotion not only reflects environmental information but also is determined by the environment. Therefore, emotion has ecological rationality and plays a key role in guiding information searching, which can thus provide an effective stopping rule for search (Gigerenzer and Selten, 2002). For instance, disgust has an adaptive value since it could suggest to a human to avoid food that is harmful to health. So, it appears that the “disgust heuristic” is more effective than cognitive heuristic strategies in this condition.

Given that society refers to a specific circumstance involving various agents, Gigerenzer argued that social rationality could be regarded as a special form of ecological rationality. That is, the circumstance in which decision-makers live is to put together other agents whom they are interacting with. According to existing literature, human judgment and decision-making could be interpreted within the social structure (Gigerenzer and Selten, 2002). In this sense, social norms might function as fast and frugal heuristics that have considerable environment adaptability. Following social norms could exempt individuals from the cost–benefit analysis in the decision process. For instance, it has been suggested that fairness heuristics, as social rationality, plays a major role in maintaining cooperative relations among humans.

As mentioned above, given that idealized rationality cannot describe decision behavior in the real world, solutions have been proposed to overcome this predicament, e.g., Simon’s bounded rationality and Kahneman and Tversky’s prospect theory. Simon, Kahneman, and Tversky primarily made contribution to understanding the deficiency of infinite rationality from the perspective of cognition but rarely recognized the role of non-cognitive factors (e.g., emotions and social norms). The concept of ecological rationality, proposed by Gigerenzer, is an acknowledgment of emotion’s role in the human decision process. Since then, emotions have been attracting increasing attention in the field of decision-making. It was not until the SMH was proposed that emotions were widely recognized by decision researchers. Based on clinical observations of decision deficits, Antonio Damasio, a leading neuroscientist, formulated the SMH, an original theory about how emotions and feelings affect daily decision-making. According to the SMH, emotion, just as cognition, is a central and indispensable component in decision-making under uncertainty (Damasio, 1994, 1996). The SMH not only provides empirical support for the central claims of ecological rationality but also considerably sets the stage for the development of embodiment of mind (e.g., embodied cognition and embodied emotion) in both neurological and physiological dimensions.

## Embodied Emotion and Somatic Marker Hypothesis

As an emerging advancement in the fields of cognitive science and psychology, the embodiment of mind goes beyond the traditional approach (i.e., information processing and cognitive computation). In short, the embodiment of mind argues that the mind depends on the physiological and the neural structures of the body and the way they work, that is, the mind derives from human body structures as well as from interactions between the body and the environment (Wilson, 2002).

The embodied cognition treats the agents of cognition as naturally and biologically adaptive individuals who live in everyday settings. Moreover, it emphasizes the key role which the body plays in the development of cognitive processes—not only are the ways of cognitive processing dominated by the physical features of the body, but the contents of cognitive processing are also furnished by the body. Anyway, cognition, body, and environment are integrated (Barsalou, 2008). Therefore, it is obvious that embodied cognition and ecological rationality are the same in underlining the interaction between human and environment.

The program of embodied cognition has spawned the advent of embodied emotion. The conventional research on emotions is dominated by disembodied theories, and hence emotions exclusively are labeled as “cognition.” According to the cognitive perspective, human emotional responses are the result of their cognitive appraisal of a situation (Spackman and Miller, 2008). In this sense, emotion, just as cognition, is treated as disembodied. However, this view has been criticized on the grounds that it largely ignored body changes, as well as neural and visceral responses, which are produced along with emotions. With the development of embodied cognition, the embodiment of emotion is gradually becoming the focus in emotion-related fields. Contrary to the conventional cognitive appraisal view, embodied emotion implies that emotions are defined by the body, including the brain, that is, it is the physical structures, way of activity, and sensation and movement experience of the body that jointly cause emotion responses and experiences (Niedenthal, 2007; Niedenthal et al., 2009).

It should be noted that, unlike embodied cognition, embodied emotion has been noticed by earlier psychologists. For instance, James’s theory of emotion (also known as peripheral theories) has touched the basic assumption of the embodied emotion. According to James’s peripheral theory of emotion, emotions result from specific physiological changes in our bodies (e.g., visceral responses, facial expressions, and muscle movements), and each emotion has a different physiological basis (James, 1884, 1894). Following the pioneering works of James, Damasio (1994) further proposed the SMH, which highlights the neurobiological basis of emotion experiences as well as the significant impact of emotions on the human decision process.

It is believed that Damasio’s SMH could provide a powerful support for both embodied cognition and embodied emotion. The SMH posits that peripheral physiological changes and visceral responses as well as corresponding central nervous representations jointly exert a significant impact on the generation of emotions, and emotions in turn influence subsequent judgment and decision-making. In other words, emotions could be construed as a collection of bodily reactions and central nervous representations elicited by a specific situation. The bodily responses include visceral activities (e.g., heart rate, gastrointestinal motility, blood pressure, etc.), gland secretion, and skeletal muscle movement. These reactions could be represented by brain regions (e.g., brainstem, insula, somatic cortex, etc.) and finally produce emotion signals, namely, somatic maker. Simply put, somatic makers not only cause emotion experience but also further affect the human decision process (Damasio, 1994, 1996).

In addition, the SMH postulates that cognitive appraisal acts as the mediator between body consciousness (emotion experience) and subsequent behaviors. Therefore, decision-making can be better understood by considering the combined effects of cognitive appraisal and body consciousness (Damasio, 1994). So, Damasio’s SMH elaborates the physiological and the neural basis of emotion based on neurophysiology, thus providing a strong empirical and theoretical support for embodied emotion. What is more, the SMH reinforces, both theoretically and empirically, the embodiment of emotion and hence contributes to the advancement of embodied mind research, which finally nurtures the frontier cross-disciplines such as cognitive neuroscience, affective and social neuroscience, and neuroeconomics.

## Neuroeconomics and Somatic Marker Hypothesis

Neuroeconomics, as an emerging research field, tries to link economics, psychology, and neuroscience to better understand economic and financial decision-making. Neuroeconomic research largely benefits from behavioral decision-making, which aims at describing and interpreting the human decision process so as to help people make appropriate decisions. As a result of the ground-breaking works of some researchers in behavioral decision-making and behavioral economics, e.g., Simon, Kahneman, Tversky, Richard Thaler, etc., neuroeconomics has emerged at the start of this century (Glimcher et al., 2008).

As it has shown repeatedly, emotions have no place in the early full rationality model of utility-maximizing decision. Bounded rationality, too, undervalues the role of emotions in judgment and decision-making (Simon, 1997). Indeed emotions have gotten researchers’ recognition such as in Kahneman and Tversky’s prospect theory. However, the prospect theory merely regards emotions as a “fast and negative” component of a dual-system processing, which may cause various decision-making biases, such as the framing effect (Kahneman and Tversky, 1979; Tversky and Kahneman, 1981; Kahneman, 2011). The multiple roles of emotions (positive or negative) were not fully discussed until neuroeconomics emerged in the recent decades. With the help of electrophysiological methods [e.g., event-related potentials, skin conductance responses (SCRs)] and brain imaging techniques [e.g., “functional magnetic resonance imaging (*f*MRI)], the joint effects of emotion and cognition on decision-making have been well documented. What is more, the SMH, as one of the foundation stones of neuroeconomics, first recognized that emotion, just as cognition, plays an indispensable and positive role in the human decision process and further showed that there is a significant interaction between emotion and cognition (Damasio, 1994; Bechara et al., 2000).

Unlike traditional economics, neuroeconomic research does not exclude the role of emotions in the decision-making process (Glimcher et al., 2008). Moreover, we believe that the SMH could be treated as a pioneering theory of neuroeconomics since it not only established a disciplinary benchmark for neuroeconomics from a neurobiological perspective but also could nourish neuroeconomics with methodological inspiration and theoretical guidance (Lo and Repin, 2002). A key assumption underlying the SMH is that emotional signals (namely, somatic marker, e.g., heart rate, blood pressure, etc.) could facilitate cognitive processing during decision-making (Damasio, 1994). According to the SMH, alternatives might be marked with a “good” or a “bad” tag by visceral responses, which in turn helps the decision-maker to make choices. The neural basis behind the aforementioned decision-making process lies in the function of the ventromedial prefrontal cortex (vmPFC). Notably, damage to the vmPFC would cause decision deficits. It is indicated that patients who have suffered damage to the vmPFC fail to produce any anticipatory SCRs before their selection of a disadvantageous option. That is to say, decision deficits result from the anticipatory somatic responses that fail to be produced, which further hinder the arousal of relevant emotion (Bechara et al., 1994). Given that these patients’ intellectual and understanding abilities are generally well preserved, it has been hypothesized that their deficit lies in an inability to make use of emotions to guide in their decision-making (Damasio, 1994). This hypothesis has been supported by existing studies based on the Iowa Gambling Task and *f*MRI (Bechara and Damasio, 2005; Chiu et al., 2018). According to a systematic review published by Reimann and Bechara (2010), collective evidence indicated that not only vmPFC but also other emotion-related brain regions, e.g., orbital frontal cortex (OFC) and amygdala, are the brain localization and neural bases underlying the effect of emotions on decision-making (Bechara et al., 1999, 2000).

As a pioneering theory concerning the relationship between emotion and decision-making, the SMH has extended, beyond the conventional topics involving decision deficit and brain lesion, into other fields, e.g., drug abuse and substance addiction (Verdejo-García and Bechara, 2009), pathological gambling (Brevers et al., 2013), anti-social personality and criminality (Sobhani and Bechara, 2011), and economic and financial decision-making (Shiv et al., 2005), and shown good adaptability. For example, based on the SMH, the anatomy and the functional organization of vmPFC have been used to provide neurobiological interpretations for consumers’ decision bias, such as delay discounting (the tendency to prefer smaller, sooner rewards to larger, later ones), i.e., gaining $1000 tomorrow is preferred to gaining $2000 2 years from now. According to the SMH, this is because “near future” is processed by posterior vmPFC, and it triggers stronger somatic responses and therefore exerts a stronger bias on decisions relative to the “distant future” (Bechara and Damasio, 2005). In sum, with the rise of decision neuroscience and neuroeconomics at the beginning of this century, the SMH has become a pioneer neurobiological theory which describes and explains the relationship between emotion, decision-making, and the brain.

Indeed despite the fact that early research on somatic markers has ultimately resulted in the publication of a vast amount of literature relating to the subject and even spurred the subsequent generation of affective neuroscience and neuroeconomics (Glimcher et al., 2008; Reimann and Bechara, 2010), the SMH has also been critiqued theoretically and empirically for overrating the influence of somatic feedback on high-order cognitive processes (Maia and McClelland, 2004, 2005; Dunn et al., 2006). This challenge may be addressed by the increasing evidence that supports the interactions between bodily states and cognitive functions (Dunn et al., 2010; Poppa and Bechara, 2018).

## Future Direction: Cross-Disciplinary Integration

### SMH Bridges Eco-Rationality, Embodied Emotion, and Neuroeconomics

Ecological rationality emphasizes the match between human and environment, as well as the crucial role of emotion and social norm in decision-making. The SMH not only provides neurobiological support for ecological rationality but also could be used as a bridge to apply ecological rationality to neuroeconomics. This is because, besides the traditional focus on emotion and decision-making, the SMH has been expanded into other topics, such as fairness preference in the Ultimate Game (Ohira, 2010). Moreover, existing evidence has indicated that the SMH not only could be used to understand issues concerning decision-making under uncertainty (van’t Wout et al., 2006) but also demonstrated that gut feelings that are highlighted by both ecological rationality and the SMH play a significant role in social decision-making (Dunn et al., 2010, 2012; Lenggenhager et al., 2013; Sokol-Hessher et al., 2015; Verweij et al., 2015). Therefore, future neuroeconomic research is expected to further explore the multi-mechanisms of specific emotions and social norms (e.g., trust, reciprocity, altruism, etc.) in economic decision-making and consumer choices.

As a high-ranking cognitive process, the embodiment of decision-making has been attracting increasing attention (Oullier and Basso, 2010; Dunn et al., 2012). For instance, Reimann et al. (2012) explicitly stated that the SMH could work as a theoretical forerunner for investigating the embodiment of judgment and decision-making because the SMH is, to date, the unique comprehensive neurobiological theory that describes the joint effect of emotion and cognition on judgment and decision-making. More importantly, the research advancement of the SMH over the past two decades has constituted a solid empirical support for the embodiment of emotion (Poppa and Bechara, 2018). Hence, the SMH is expected to offer theoretical and methodological implications to future researchers who are interested in applying embodied emotion to neuroeconomics.

What is more, the most important consensus between ecological rationality, embodied mind, and the SMH is that human adaptability to the environment and the evolution of the body and the brain play a crucial role in human cognition and decision-making process. In this case, the SMH can serve as a connection bridge between ecological rationality, embodied emotion, and neuroeconomics for exploring the respective role of rationality and emotion in decision-making under uncertainty (Chiu et al., 2018).

### Integrating Eco-Rationality, Embodied Emotion, and Neuroeconomics Under the SMH

Interestingly, both Gigerenzer and Damasio underline the positive role of gut feelings in judgment and decision-making from both evolutional and ecological perspectives. In their view, the full rationality would not only be impossible to achieve but also not be the only solution (Damasio, 1994; Gigerenzer, 2000). The rule of thumb and intuitive judgment accumulated through evolution and adaptation may be more efficient than logical analysis and rational calculation to solve practical problems, that is, “ignorant hunch” is sometimes superior to “knowing calculations.” For instance, the “less is more” of recognition heuristics is often more reliable than the “more is better” of maximizing decision in the real world (Gigerenzer, 2007; Damasio and Carvalho, 2013). In this sense, like the SMH, ecological rationality could also be introduced to explore the neurobiological mechanisms of economic decision-making and consumer choice.

According to the SMH, intuition or gut feelings may be a far more accurate predictor under uncertainty relative to market data and fact lists (Bechara and Damasio, 2005). For this, the SMH offers an explanation for how decision-makers make a proper decision based on gut feelings. Especially the striatum (Str) and the anterior cingulate (ACC) of the human brain are involved in pattern recognition and probability calculation, and these two brain regions could give immediate response to the repetition of patterns. The Str and ACC would be able to make accurate predictions when market volatility is a reliably simple repetition and alternative model (Reimann and Bechara, 2010). Conversely, if information is complex and pattern is ambiguous, somatic state, which manifests as presentiment or gut feeling, could help us to choose the most optimal alternative despite the fact that cognitive computing and deliberation can also provide a certain choice. Once the somatic state is activated by primary and/or secondary inducers, an overall positive or negative somatic state then appears. Furthermore, the development of the overall somatic state is subject to the law of natural selection, that is, the stronger somatic signals would vanquish the weaker signals, until finally an overall somatic state (i.e., presentiment and gut feelings) that achieves a dominant position occurs, which in turn exerts influence on subsequent decision behavior (Bechara and Damasio, 2005; Damasio and Carvalho, 2013). In sum, the SMH provided robustly empirical support for the theoretical view of ecological rationality as proposed by Gigerenzer (2000, 2007).

In addition, investigating the relationship between embodied emotion and the neurobiological mechanism of economic decision-making is in line with the current trend of interdisciplinary integration, that is, embodied emotion and neuroeconomics complement each other. On the one hand, neuroeconomic research concerning the neural mechanism of economic decision-making could provide neurobiological support for the embodied mind (cognition and emotion). More specifically, cognitive and emotional processing in the brain are synergistic rather than separate, and vmPFC and OFC are just the neural basis of the interaction of both cognition and emotion. Neuroeconomic research, as well as decision neuroscientific research, has repeatedly proved that humans’ cognitive processing is susceptible to their emotion and that purely disembodied cognition and independent rational brain regions that are divorced from emotion do not exist (Glimcher et al., 2008). Moreover, the artificial divisions that we have made between cognition and motivation, as well as rationality and emotion, merely result from human-phrased knowledge. Also, existing literatures on the brain mechanisms of cognitive neuroscience, affective and social neuroscience, have suggested that there are no specific brain regions that could independently perform a single mental function. In sum, different brain regions are interconnected, synergistic, and together functioning (Glimcher and Fehr, 2014). On the other hand, the embodiment of mind (cognition and emotion) could provide neuroeconomic studies on decision-making with theoretical and methodological guidance. Not only this, the SMH could act as a bridge between the embodiment of mind and the brain mechanism of economic decision-making. In this case, neuroeconomics, as a frontier cross-discipline which combines economics, psychology, and neurosciences, is expected to benefit from the embodied mind and to contribute to the advancement of research on embodied emotion and ecological rationality, which will in turn further promote emerging decision neuroscience and consumer neuroscience that have been derived by the SMH (Oullier and Basso, 2010; Reimann et al., 2012; Verweij et al., 2015).

## Conclusion: An Integrated Framework of Rationality, Emotion, and Neuroeconomics

**FIGURE 1:**
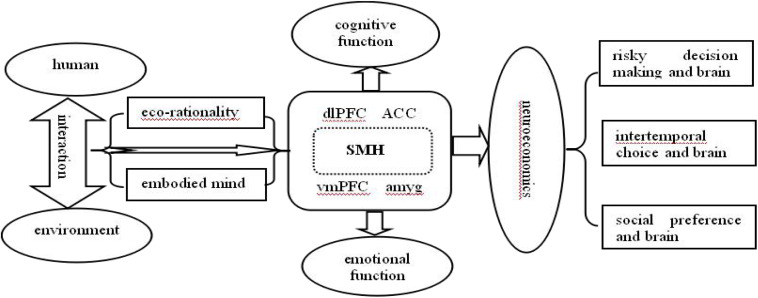
An integrated framework for applying ecological rationality and embodied mind to neuroeconomics under the somatic marker hypothesis.

Based on the above-mentioned discussions, an integrated framework could be presented. As shown in Figure 1, humans have evolved a sophisticated nervous system while interacting with the environment, and the interactive relationship between humans and their environment is constantly being updated at the same time, which finally sets human ecological rationality and embodied mind. Furthermore, the SMH, as a pioneer theory of neuroeconomics, provides comprehensive evidences and explanation for ecological rationality and embodied emotion. It is believed that human decision-making involves not only cognitive processing (e.g., executive functions and working memory) produced by dlPFC, ACC, etc., but also emotional processing (e.g., emotions and feelings) produced by vmPFC, amygdala, etc. Taking these into account, future neuroeconomic research, particularly concerning risky decision-making, intertemporal choice, or social preference, is expected to take advantage of the insights provided by the SMH, ecological rationality, and embodied mind to gain a better understanding of economic decision-making and its neurobiological mechanism.

Lastly, this prospect could be illustrated with the example of fairness preference. As a kind of social preference, fairness preference manifests that people are sensitive to benefit, as well as the fairness of benefit distribution. In fact, our preference for fairness is closely related to our value on social norms. According to ecological rationality, preference for fairness could be viewed as a fairness heuristic which is established on the basis of social norms, that is, in order to maintain social structure and interpersonal relationship, people tend to make a decision that is desired by members of the same social status (Gigerenzer and Selten, 2002). More importantly, existing neuroeconomic research has repeatedly proved that vmPFC, anterior insula, dlPFC, as well as ACC (which is associated with conflict monitoring) jointly constitute the neural pathways of fairness preference. This suggests that cognition and emotion are indispensable to fairness preference. In addition, research on fairness preference using an electrophysiologic approach has shown that unfair distribution (*vs*. fair distribution) could elicit larger feedback-related negativity amplitudes, higher SCR, and smaller heart rate. The neurophysiological basis of fairness preference is in line with the tenet of the SMH (Naqvi et al., 2006; Ohira, 2010). In this sense, applying ecological rationality and embodied emotion into future neuroeconomic research by virtue of the SMH will help advance our knowledge of human rationality, emotion, and decision-making.

## Author Contributions

FX conceived and designed the review. LH and FX wrote the manuscript. PX and FX revised the manuscript.

## Conflict of Interest

The authors declare that the research was conducted in the absence of any commercial or financial relationships that could be construed as a potential conflict of interest.
